# IgG antibody responses to *Plasmodium falciparum *merozoite antigens in Kenyan children have a short half-life

**DOI:** 10.1186/1475-2875-6-82

**Published:** 2007-06-28

**Authors:** Samson M Kinyanjui, David J Conway, David E Lanar, Kevin Marsh

**Affiliations:** 1 Kenya Medical Research Institute, Centre for Geographic Medicine Research-Coast, P.O. Box 230, Kilifi, Kenya; 2 Department of Infectious and Tropical Diseases, London School of Hygiene and Tropical Medicine, WC1E 7HT, London, UK & MRC laboratories, Fajara, P.O. Box 273, Banjul, The Gambia; 3 Department of Immunology, Walter Reed Army Institute of Research, Silver Spring, MD 20910, USA

## Abstract

**Background:**

Data suggest that antibody responses to malaria parasites merozoite antigens are generally short-lived and this has implications for serological studies and malaria vaccine designs. However, precise data on the kinetics of these responses is lacking.

**Methods:**

IgG1 and IgG3 responses to five recombinant *Plasmodium falciparum *merozoite antigens (MSP-1_19_, MSP-2 type A and B, AMA-1 ectodomain and EBA-175 region II) among Kenyan children were monitored using ELISA for 12 weeks after an acute episode of malaria and their half-lives estimated using an exponential decay model.

**Results:**

The responses peaked mainly at week 1 and then decayed rapidly to very low levels within 6 weeks. Estimation of the half-lives of 40 IgG1 responses yielded a mean half-life of 9.8 days (95% CI: 7.6 – 12.0) while for 16 IgG3 responses it was 6.1 days (95% CI: 3.7 – 8.4), periods that are shorter than those normally described for the catabolic half-life of these antibody subclasses.

**Conclusion:**

This study indicates antibodies against merozoite antigens have very short half-lives and this has to be taken into account when designing serological studies and vaccines based on the antigens.

## Background

An effective malaria vaccine is urgently needed, but to date it remains elusive. A common way of trying to establish if a given malaria parasite antigen is a good candidate for a malaria vaccine is by determining if an with protection against subsequent infections of malaria. However, a number of studies suggest that naturally acquired responses to malaria merozoite antigens are short-lived. Among the majority of people living in endemic areas, levels of antibodies to merozoite antigens appear to vary with the levels of malaria transmission i.e. they are highest during periods of intense transmission and lowest or undetectable at the end of periods of low transmission [1-3]. Further, levels of antibodies to merozoite antigens often tend to be higher in individuals who also have malaria parasites at the time when their antibodies are measured than in those without parasites [2,4-6].

The implication of these observations is important as they suggests that during serological surveys, individuals who can nonetheless mount a rapid secondary antibody response to malaria antigens upon re-infection are likely to be classified as antibody negative depending on how recent their last malaria infection was. Conversely, individuals who are positive at the survey may be negative by the time they encounter the next infection. If indeed the antibodies responses are very brief, then data from longitudinal studies with long intervals between sampling days will not reflect well the dynamics of the responses. Unfortunately, estimates of the half-lives of antibody responses to malaria that can help guide the design of such studies are lacking. In this study, a closely spaced sampling schedule was used to monitor the kinetics of antibody responses to five recombinant *Plasmodium falciparum *merozoite antigens among Kenyan children recovering from a clinical infection of malaria and the data used to estimate the half-life of the responses. The results of the study indicated that both IgG1 and IgG3 antibodies to merozoite antigens have very short half-lives.

## Methods

### Study population and blood sampling

This study was carried out at Kilifi District Hospital (KDH) on the Kenyan coast. Ethical clearance for the study was given by the Kenya Medical Research Institute ethics review board. Forty eight children admitted to the pediatric ward of KDH with a primary diagnosis of malaria, but who did not fulfill the World Health Organization criteria for severe malaria [7], were recruited, if their guardian gave written consent. A venous blood sample was taken from each child at recruitment and, subsequently, at as many of the time points as possible 1, 2, 3, 6, 9, and 12 wks after treatment with sulphadoxine/pyrimethamine (SP). The samples were centrifuged at 700 × *g *for 5 min to obtain plasma, which was stored at 20°C. The children were examined by a clinician and a thick malaria film prepared during the follow-up visits or any other time during the study when they were unwell. Malaria treatment (SP) was given for parasitaemia in the presence of fever (axillary temperature ≥ 37.5°C). Seven children from whom weeks 1 and 2 samples could not be obtained were considered lost to follow up, so the cohort for analysis comprised 41 children.

### ELISA

IgG1 and IgG3 antibody reactivity to recombinant ectodomain of *P. falciparum *apical merozoite antigen 1(AMA-1), the 11 kDa carboxyl portion of merozoite surface antigen 1 (MSP-1_19_), region II of the 175 kDa erythrocytes binding antigen (EBA-175 RII), and two recombinant proteins representing the two major allelic types of MSP-2 was assessed in plasma samples from 41 children (age range = 7 – 107 months, median = 34 months). Levels of IgM reactivity against the two allelic types of MSP-2 were also assessed. The AMA-1 antigen and EBA-175 region II were kind gifts from Sheetij Dutta and Arnoldo Barbosa (WRAIR, Maryland, USA) and have been previously described [8,9], while MSP-1_19 _and MSP-2 proteins were provided by Jana McBride and David Cavanagh (University of Edinburgh, UK) and have also been previously described [10,11]. Plasma was assayed at 1:4000 dilution for antibodies to AMA-1 and at 1:500 for antibodies to the other antigens using ELISA protocols described previously [3,11,12]. Briefly, the wells of 96-well plates (Immulon4; Dynatech, Chantilly, VA) were coated overnight at 4°C with either 50 ng (MSP-1_19 _and MSP-2) or 100 ng (EBA-175 and AMA-1) of antigen in sodium carbonate buffer (pH 9.3), washed, and then blocked with 1% skimmed milk in coating buffer for 5 h and washed again. The wells were subsequently incubated overnight at 4°C with 100 μl of plasma diluted in 1% skimmed milk, followed by a three-hour incubation with 100 μl of horseradish peroxidase-conjugated rabbit antibodies against human antibodies (Dako Ltd, Cambridge, UK) at 1/1000 for anti-human IgG1 and IgG3 or 1/5000 for anti-human IgM, with washing between the incubations. Colour was developed with *o*-phenylenediamine, the reaction stopped after 15 minutes with 20 μl of 2 M H_2_SO_4_, and absorbance was measured as optical density (OD) at 492 nm. All samples were simultaneously assayed in duplicate for IgG1 and IgG3 antibodies against a given antigen. Absorbance readings for responses against MSP-1_19 _and MSP-2 antigens were corrected for responses to the GST fusion carrier by deducting the absorbance of the same plasma assayed against GST alone. The cut-off for absorbance readings for each antigen was set as mean plus three standard deviations of absorbance readings of sera from eight Europeans without any history of malaria infection. In order to convert absorbance to concentration (μg/ml), plates were coated with purified human myeloma proteins IgG1, and IgG3 (at threefold serial dilutions from 10 to 5.1 × 10^-4 ^μg/ml).

The resulting standard absorbance versus concentration curves were used for interpolation of the absorbances of the plasma samples to estimate concentrations.

### Estimating the half-life of the responses

To estimate the half-life of antibody responses to the test antigens, response profiles that showed a decline of antibody levels over at least four consecutive sampling points were selected and the decay phase analysed up to the sampling point where the ODs were too low for concentration to be estimated from standard curves, or the decline was obviously interrupted by boosting. After conversion of absorbance to concentration as described above, one-phase exponential decay curves defined by the equation below were fitted to the decay phase of the selected response profiles using Graphpad Prism software ver. 3.2 (San Diego, USA).

[C]_t _= [C]_0_e^-kt ^+ Plateau

Where [C]_0 _and [C]_t _are concentrations at time 0 and t respectively, k is a rate constant and plateau is the concentration at the end of the decay and half-life of the decay (t_1/2_) is calculated as follows:

ln⁡12[C]0[C]0=−kt12  therefore t12=ln⁡2k
 MathType@MTEF@5@5@+=feaafiart1ev1aaatCvAUfKttLearuWrP9MDH5MBPbIqV92AaeXatLxBI9gBaebbnrfifHhDYfgasaacH8akY=wiFfYdH8Gipec8Eeeu0xXdbba9frFj0=OqFfea0dXdd9vqai=hGuQ8kuc9pgc9s8qqaq=dirpe0xb9q8qiLsFr0=vr0=vr0dc8meaabaqaciaacaGaaeqabaqabeGadaaakeaacyGGSbaBcqGGUbGBdaWcaaqaamaalmaaleaacqaIXaqmaeaacqaIYaGmaaGccqGGBbWwcqqGdbWqcqGGDbqxdaWgaaWcbaGaeGimaadabeaaaOqaaiabcUfaBjabboeadjabc2faDnaaBaaaleaacqaIWaamaeqaaaaakiabg2da9iabgkHiTiabbUgaRjabbsha0naaBaaaleaadaWcdaadbaGaeGymaedabaGaeGOmaidaaaWcbeaakiabbccaGiabbccaGiabbsha0jabbIgaOjabbwgaLjabbkhaYjabbwgaLjabbAgaMjabb+gaVjabbkhaYjabbwgaLjabbccaGiabbsha0naaBaaaleaadaWcdaadbaGaeGymaedabaGaeGOmaidaaaWcbeaakiabg2da9maalaaabaGagiiBaWMaeiOBa4MaeGOmaidabaGaee4AaSgaaaaa@5A67@

### Assessing factors that might influence the kinetics and half-life of responses

Association between kinetics half-life of the responses and age of responder or peak level of antibodies at the beginning of the decay was assessed by linear regression using Stata ver. 8 (Stata Corporation, TX, USA), while differences between half-lives of response to the various test antigens was assessed using one-way ANOVA. In addition, ANOVA was used to test if the mean level of responses to the test antigens differed between individuals. The profiles were then grouped together depending on whether or not they had peaked before the child presented to hospital, and the mean half-lives of the groups compared using a student t-test.

## Results

### Antibody responses to *P. falciparum *antigens in the study children

**Figure 1:**
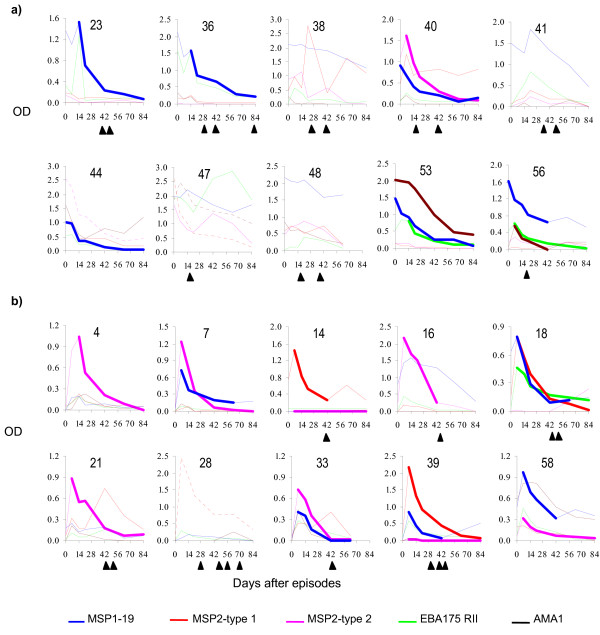
**Examples of  absorbance profiles of IgG1 response to the test  antigens among the study children**.  Day 0 is the day of presentation to  hospital.  The number above each plot is the child's study number.  a)  Children who had moderate to high level of antibodies to one or more  antigens at presentation to hospital.  b) Children who made a distinct  responses to one or more antigens after presentation to hospital.  For  the responses whose half-life was estimated, the sampling points used in  the estimation are joined by a bold line.  Responses that peaked beyond  the interpolation range of the standard curve are plotted in dotted  lines.  The solid triangles below the plots indicate time points when  the children were positive for malaria parasites.

**Figure 2:**
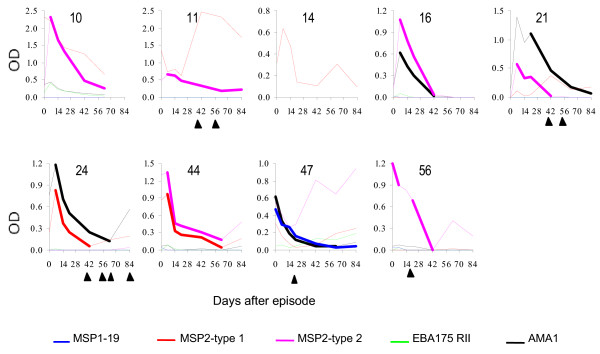
**IgG3 antibodies responses to the test antigens among children  where some of the profiles were used in half-life estimation.  **Day 0 is  the day of presentation to hospital.  The number above the plot is the  child's study number.  The sampling points used in half-life  estimation are joined with a bold line.  The solid triangles below the  plots indicate time points when the children were positive for malaria  parasites.

There was considerable variation in the children's overall pattern of responses to the five test antigens but for each antigen, the children generally responded in one of three ways. Either, (1) they already had moderate to high levels of antibodies to the antigen by the time they presented to hospital (e.g. IgG1 responses to MSP-1_19 _in subjects 23, 36, 38, 40, 41,47, 48 and 56, Figure 1a; IgG3 response to MSP-2 in subjects 10, 44, and 56, Figure 2) or, (2) they presented with low levels of antibodies but made sharp responses that peaked one week after the episode and then declined rapidly (IgG1 responses to MSP-2 in the plots in Figure 1b) or, (3) they made only very low responses (many of the responses plotted in Figures 1a and 1b and Figure 2). IgM antibodies to the two MSP-2 antigens was also assayed to see if the children were making primary responses. Only in two subjects did the IgM absorbance rise above the cut off value during the study period: in subject 28 anti-MSP-2 type B IgM peaked at week one with an absorbance of 0.74, which was much lower than that of the strongest IgG responses that the child made (absorbance of 2.42 for IgG1 to MSP-2 type A); child number 44 had anti-MSP-2-type B IgM absorbance of 0.74 at presentation, which was also much lower than the strongest IgG response (absorbance of 2.55 for IgG1 to MSP-2 type B).

### Boosting of responses over time

After peaking, the responses generally showed a steady decline but eventually some boosting was observed. Boosting of responses to individual antigens was often associated with the appearance of malaria parasites in the IgG3), but not always (in 15 instances for IgG1, and six for IgG3). Only in 10 of the 43 individual antigen-specific boosting instances were IgG1 responses boosted to similar or higher levels than the initial peak (for example, anti-MSP-2 type A response in subject 38, anti-EBA-175 response in subject 47, Figure 1a), and only five of 15 instances did such boosting occur in the case of IgG3 responses (e.g. anti-MSP-2 type B response in subject 47, Figure 2). 28/41 (68%) children became positive for malaria parasites at one or more sampling time points, mainly 5 weeks or longer after the clinical episode.

### Response profiles selected for half-life estimation

Sixty nine IgG1 response profiles from 28 children showed monophasic decay over four consecutive time points. Of these, 40 profiles from 23 children had four consecutive sampling points with absorbance within the interpolation range of the standard curves and were used in the half-life estimation. Of the 26 IgG3 response profiles from 15 children that exhibited monophasic decay over four consecutive time points, 16 profiles from nine children had absorbance within interpolation range of the standard curve, and were used for analysis of half-life.

### Half-life of antibody responses to the test antigens

**Figure 3:**
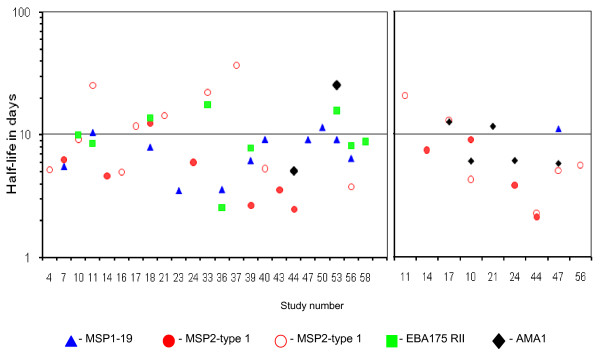
**Half-lives of individual IgG1 and IgG3 responses to the test antigens in days**. Among some children, response profile to only one of the four test antigens fulfilled the criteria for half-life analysis while among the other children, profiles of responses to two or more antigens were analysed.

The exponential decay model used had a fit of R^2 ^> 0.90 for the profiles analysed. The mean half-lives of the 40 IgG1 responses analysed individually was 9.8 days (95% confidence interval [CI]: 7.6 – 12.0). There was no association between half-life and age (r^2 ^= 0.055, P = 0.130), peak level of antibody (r^2 ^= 0.003, P = 0.748), or antigen specificity (F = 0.547, P = 0.653). The half-lives of the responses did not differ significantly between different individuals (F = 2.198, P = 0.140; ANOVA on data from five individuals for each of whom the half-lives of responses to three antigens were estimated). The mean half-life of the IgG3 responses analysed was lower (6.1 days, 95% CI: 3.7 – 8.4) than that of IgG1 but not significantly (Mann-Whitney U test, P = 0.424). As with IgG1, there was no association between half-life and age (r^2 ^= 0.13, *P = 0.210*), peak levels of antibody (r^2 ^= 0.74, *P = 0.273*), or antigen specificities (F = 0.448, *P = 0.647*) (Figure 3). For both isotypes, the small number of data points analysed for each profile did not allow reliable estimation of the confidence intervals of the half-lives within each individual child.

### Half-life of responses in relation to status at presentation to hospital

The profiles were grouped depending on whether or not they had peaked by the time the child was presented to hospital and compared their mean half-lives. IgG1 profiles which peaked before presentation to hospital (n = 8) had a longer mean half-life (13.1 days [95% CI: 8.2 – 21.4]) than those that peaked after presentation (*n = 32*) (8.9 days [95% CI: 5.5–10.8]) but the difference was not significant (P = 0.17). The mean half-life of IgG3 responses that peaked before presentation to hospital (*n = 5*) was 7.3 days (95% CI: 5.0 9.5), while that of the responses that peak post presentation (*n = 10*) was 8.1 days (95% CI: 4.8 – 11.5), a small difference that was not significant (P = 0.685)

### Correlation between half-life of responses within the same child

Data from 11 children where responses to two or more antigens were analysed for half-life was used to determine the correlation between the half-life of responses to different antigens within the same child. A high level of correlation between responses to different antigens by the same child was observed (R^2 ^= 0.63, P = 0.004).

## Discussion

Estimating the half-life of antibody responses to malaria antigens among residents of malaria endemic areas is difficult as the kinetics of the antibodies are continually altered by reinfection. In this study, eventual boosting of the initial responses was evident in many of the children even in the absence of patent re-parasitization. As such, half-lives could only be estimated for those responses that showed no obvious boosting over at least four time points. However, it is still possible that the half-life estimates obtained may still have been subtly affected by sub-patent of re-parasitization. In addition the half-life of the some of the profiles that were not analysed because they did not meet the inclusion criteria may have been different from those estimates obtained but it was not possible to clarify this. As such, a future study is being planned that will include a group of children who are kept parasite-free over study period by chemoprophylaxis. Nevertheless, this study shows that among Kenyan children recovering from clinical malaria, antibody responses to *P. falciparum *merozoite antigens particularly IgG1 often have very short half-lives. While the estimated mean half-life of the IgG3 antibodies (6 days) was similar to that of antibodies injected into healthy adults (8 days) [13], that of IgG1 (9.8 days) was about half that of the exogenous antibodies (21–23 days).

Such short half-lives could explain observations from previous longitudinal studies that reported a rapid decline of antibodies to malaria antigens within a few months of malaria treatment. For instance, 44% of Brazilian individuals who made antibodies to the C-terminal fragment of *P. vivax *merozoite surface protein 1 (MSP-1_19_) during a clinical episode of malaria became sero-negative within two months of treatment [6]. Similarly, antibodies to *P. falciparum *MSP1 and rhoptry associated protein 1(RAP-1) in Sudanese individuals often last for less than three months after treatment for clinical malaria [3,14].

Data on the half-lives of natural responses to other infectious agents such as bacteria are generally lacking and it is not possible to say if the short-lives observed here are unique to malaria or common to acute infection by different pathogens. Future studies will have to incorporate patients with other conditions for comparison. On the other hand, antibodies responses to viral infections and vaccines such as measles, appear to last for many years [15,16] although possibly sustained by persistent cryptic viral particles [17]. Interestingly, malaria chemoprophylaxis was associated with a slower rate of decay of antibodies to a meningococcal vaccine among Gambia children [18] suggesting that acute malaria might affect the longevity of antibodies universally.

Although the half-life of IgG1 is usually cited as 21 days [13], early studies showed the half-life is inversely related to serum concentration and for IgG1, IgG 2 and IgG 4 it may vary from over 70 days to 11 days [19]. Malaria episodes are associated with raised concentrations of both specific and polyclonal of antibodies [20-22] and this may, in part, explain the reduced IgG1 antibody half-lives observed. However, in some children, the antibodies had extremely short half-lives suggesting that other processes, besides ordinary catabolism, may be contributing to the decay. The clearance of immune complexes whose levels and rate of clearance by macrophages are raised during acute episodes of malaria [23,24] is a possible other process, but its contribution is not clear currently.

Since in natural responses, the kinetics of antibodies is a balance between production and decay the profiles observed in this study suggest that production of antibody against merozoite antigens is not sustained following an acute episode of malaria. Antibody production can be sustained through re-stimulation of memory B cells by persistent antigens [25,26] or by non-proliferating long-lived plasma cells [27,28]. In mice, long-lived plasma cells survive mainly in the bone marrow [28-30] and have a half-life of about five months [31], compared to a lifespan of 3–5 days for short-lived plasma cells [29]. Thus, the kinetics of the antibody responses described here are consistent with production by short-lived plasma cells. Clearly further studies on the effects of acute episodes of malaria on commitment of plasma cells to the memory or long life compartment is required. For example, how does the adherence of red cells infected with either sexual or mature asexual stages of malaria parasites to bone marrow stromal cells [32] affect the survival of long-lived plasma cells?

Interestingly, in many instances, re-parasitization after the episode did not boost the initial responses or only did so mildly. While polymorphism in the new infection might explain some of the failure, the fact that boosting occurred in many instances but only to a lower level than the initial responses suggests that there may be a period of suppression of antibody responses following a clinical episode of malaria. Reduced reactivity to viral vaccines during malaria episodes and recovery of reactivity following malaria treatment has been reported among Nigerian children [33].

## Conclusion

This study shows that following an acute episode of malaria in children living in an endemic area, antibody responses to malaria merozoite often have very short half-lives; less than those reported for normal antibody clearance. This has implication for studies monitoring responses to these antigens and indicates the need for closely spaced sampling within the first few weeks of an episode. Further studies on the fate of antibody producing cells following a malaria episode are required in order to elucidate the mechanism underlying this brevity. In addition, it is important to establish whether the brevity of the responses is due to malaria disease processes or whether it is inherent to the antigens. Would vaccines based on the antigens also induce short-lived responses in healthy children, and would this compromise their use, or would memory B cell populations be adequate for a sufficient antibody response upon infection?

## Competing interests

The author(s) declare that they have no competing interests.

## Authors' contributions

SMK carried out longitudinal sampling, the ELISA and prepared the manuscript DJC developed the ELISA and helped in the manuscript preparation DEL participated in the preparation of the antigens used in the study KM helped in designing the study and setting up of the longitudinal framework
